# Adherence to interferon β-1a therapy using an electronic self-injector in multiple sclerosis: a multicentre, single-arm, observational, phase IV study

**DOI:** 10.1186/s13104-016-1948-z

**Published:** 2016-03-08

**Authors:** Virginia A. Devonshire, Anthony Feinstein, Patrick Moriarty

**Affiliations:** Department of Neurology, University of British Columbia, Vancouver, Canada; Department of Psychiatry, Sunnybrook Health Sciences Centre, University of Toronto, Toronto, Canada; A division of EMD Inc., EMD Serono, 2695 North Sheridan Way, Suite 200, Mississauga, ON L5K 2N6 Canada

**Keywords:** Relapsing multiple sclerosis, Disease-modifying drugs, Adherence, Cognitive impairment, Injection anxiety

## Abstract

**Background:**

In a multicentre, single-arm, observational, phase IV study, we evaluated 24-week treatment adherence of relapsing multiple sclerosis (RMS) patients using an electronic auto-injection device (RebiSmart^®^) for subcutaneous injection of interferon (IFN) β-1a.

**Methods:**

A total of 162 adult participants with RMS were enrolled into the study to use RebiSmart^®^ to self-administer IFN β-1a 44 μg three times weekly for a maximum of 96 weeks. The number of administered injections was recorded in the electronic device log. Adherence to treatment was defined as the administration of ≥80 % of expected injections. Cognitive impairment and injection anxiety were assessed via questionnaires.

**Results:**

Overall, 91.8 and 82.9 % of participants were adherent to treatment at weeks 12 and 24, respectively. By weeks 12 and 24, 8.2 and 13.9 % of participants had discontinued treatment. There were no statistically significant differences in adherence rates at weeks 12 and 24 according to cognitive impairment status or injection anxiety. By week 24, 69.9 % of participants were less fearful of injection than when they started the study. According to participant evaluations, the absence of a visible needle, comfort settings, and the calendar for tracking the injection schedule were all important features of the RebiSmart^®^ injection system. At week 24, 99.3 % of participants reported that they would like to continue using RebiSmart^®^ as their injector.

**Conclusions:**

RebiSmart^®^ use is associated with high treatment adherence, as objectively assessed using electronic injection logs. Future research should examine if RebiSmart^®^ use improves long-term treatment outcomes in RMS.

*This study was registered with ClinicalTrials.gov as NCT01128075, on May 20, 2010.*

## Background

Multiple sclerosis (MS), a chronic inflammatory degenerative autoimmune disorder of the central nervous system, affects approximately 240 out of every 100,000 Canadians [1]. Relapsing forms of MS (RMS), including relapsing-remitting MS and secondary progressive MS, account for approximately 80 % of MS cases and are characterized by progressive disability accrued through repeated disease relapses [2, 3]. Although MS remains incurable, it can be effectively managed via proper treatment with disease-modifying drugs (DMDs) such as β-interferons [3]. When started early in the course of disease and maintained continuously, DMD treatment can reduce relapse frequency, slow disability progression, and improve long-term prognosis [4, 5].

Unfortunately, patient adherence to treatments for MS can be inconsistent, leading to poor treatment response and suboptimal long-term patient outcomes. Adherence to medication is the extent to which patients take medications as prescribed by their health-care provider [6, 7]. While there is no consensus on an acceptable rate of adherence, studies in MS have defined 80 % as a general threshold for maintaining treatment effectiveness [8]. Recent estimates suggest that 25–39 % of MS patients miss at least one injection within a 4-week period [9, 10], and only 52.2–62.3 % of MS patients are ≥80 % adherent to their injectable treatment over a 12–36-month period [8, 11]. In addition to intermittently missing treatment doses, a significant proportion of patients prematurely discontinue treatment within the first year. A recent Canadian report indicated cumulative discontinuation rates between 20.9 and 26.4 % at 6 months, 36.9 and 40.9 % at 1 year, and between 52.6 and 58.5 % at 2 years among a cohort of adult MS patients in Ontario [12]. Many patients who discontinue their initial treatment never reinitiate therapy [13]. Several studies have established that intermittently missed dosing and treatment interruptions are associated with significantly increased risks of disease relapse, hospitalization [5, 11], reduced quality of life [9], and increased health-care costs [11].

MS patients may experience barriers to treatment adherence that include forgetfulness (reported by 50.2 % of non-adherent patients) and injection-related issues (32 %) such as injection anxiety, injection pain, injection fatigue, and skin reactions [9]. Fatigue, flu-like symptoms, and headache have been reported by 10–15 % of patients as additional reasons for non-adherence [9]. These challenges may also play a role in treatment discontinuation. For instance, a poor injection experience, characterized by injection anxiety, injection pain, injection fatigue, or injection-site reactions, can lead to premature treatment discontinuation [14, 15].

Symptoms of MS may present additional barriers to treatment adherence. For example, cognitive impairment affects 40–65 % of MS patients, sometimes presenting at the earliest stages of the disease [16]. Cognitive impairment can include deficits in several cognitive domains, including memory, and may impact patients’ ability to remain adherent to their medication [17]. Adherent patients have been found to have less neuropsychological impairment, as measured by the Multiple Sclerosis Neuropsychological Screening Questionnaire, compared to non-adherent patients [9].

RebiSmart^®^, an electronic, hand-held, multidose, auto-injection device for IFN-β-1a (Rebif^®^), was designed to address the psychological and physical barriers to injection, thereby helping patients adhere to treatment [18]. RebiSmart^®^ uses a multidose cartridge that contains three doses of 44 µg IFN-β-1a and can be stored at room temperature for 4 weeks [19], making injection preparation much simpler. The injection experience is improved by incorporating a needle that is never visible to the patient and customizable injections settings, such as needle speed, injection speed, injection time, and needle depth. RebiSmart^®^ incorporates an electronic dosing log and an alarm that reminds patients of previous and upcoming injections, respectively. The dosing log captures completed, partial, and missed injections, allowing patients to see their adherence to treatment over the previous 4 weeks. The entire injection log can be uploaded to a computer, enabling physicians to objectively monitor adherence. Current research indicates that health-care professionals [20] and patients [21] routinely overestimate adherence to treatment. An objective method of measuring treatment adherence would help clinicians determine whether breakthrough disease in a given patient is due to ineffective treatment or treatment non-adherence.

The suitability of the RebiSmart^®^ device for self-injection was evaluated in RMS patients in a phase IIIb study [22]. At week 12, 71.6 % of patients considered the device “very suitable” or “suitable” for injection. The majority of patients rated “overall convenience” as the most important benefit of RebiSmart^®^. The objective of the current study was to evaluate 24-week treatment adherence in treatment-naive participants with RMS using RebiSmart^®^. We assessed whether features of the device were effective in overcoming common barriers to treatment adherence.

## Methods

### Study design

MEASURE is a multicentre, single-arm, observational, 96-week, phase IV study to evaluate treatment adherence when using RebiSmart^®^ for self-injection of subcutaneous (sc) IFN-β-1a in multidose cartridges in participants with relapsing multiple sclerosis (NCT01128075). The primary endpoint was evaluated when all participants had either completed 24 weeks of treatment or discontinued MEASURE prior to 24 weeks. Data at 48 and 96 weeks will be reported at a later date.

Participants were evaluated for study eligibility during a screening period of up to 28 days. After enrolment, each participant attended a clinic visit on study day 1 (SD1) to complete baseline assessments, receive training on RebiSmart^®^, and administer his or her first injection under the supervision of the RebiSmart^®^ trainer. Participants returned to the clinic for assessments at weeks 12 and 24 and were evaluated by telephone contact at weeks 4, 8 and 16.

Participants were provided with a RebiSmart^®^ injector for the duration of the study and were trained by a nurse on proper use of the device and on standard practice for rotating injection sites. Participants followed the IFN β-1a dose titration schedule presented in the Canadian Product Monograph: 8.8 μg tiw during weeks 1–2, 22 μg in weeks 3–4, and 44 μg tiw from week 5 onward [19]. Participants who could not tolerate the 44 μg dose following the titration period had their dose reduced to 22 μg tiw at the discretion of the treating physician. Prophylactic non-steroidal anti-inflammatory drugs could be used for the treatment of flu-like symptoms. Participants could withdraw from the study at any time. Upon a patient’s withdrawal, all assessments required at the early termination (ET) visit were completed at the earliest time possible.

### Study objectives and endpoints

The primary objective of this study was to evaluate treatment adherence over 24 weeks when using RebiSmart^®^ for self-injection of Rebif^®^ in a multidose cartridge. The primary endpoint was the proportion of participants at week 24 who were adherent to treatment, defined as having administered ≥80 % of scheduled injections as recorded in the RebiSmart^®^ injection log. A cut-off of ≥80 % was used to define adherence consistently with most clinical trials [7]. Additionally, this level of adherence appears to correlate with reduced risk of severe relapse [5]. Treatment adherence over 24 weeks was calculated as 100 × the number of administered injections recorded in the electronic device log, divided by the expected number of injections (72). Participants who discontinued treatment before week 24 were included in the analyses of 24-week adherence, with all injections scheduled after discontinuation considered missed injections.

Secondary outcome measures also assessed at 24 weeks included treatment persistence, treatment compliance, treatment adherence among cognitively impaired and non-impaired participants, longitudinal changes in injection anxiety, and qualitative participant experiences with RebiSmart^®^. Treatment persistence was calculated as the proportion of participants who remained on treatment at 24 weeks following SD1. Treatment compliance at 24 weeks was calculated as 100 × the number of administered injections recorded in the electronic device log, divided by (the number of days on-study) × 3/7. It is important to note that the calculation of 24-week treatment adherence accounts for doses missed before and after treatment discontinuation (up to week 24), and the calculation of treatment compliance accounts only for those doses missed before treatment discontinuation, for those patients who discontinued treatment prior to week 24. To determine whether impaired cognitive function was associated with poorer adherence, 24-week adherence was compared between cognitively impaired and non-impaired participants. The change in anxiety was evaluated by comparing baseline anxiety scores of the hospital anxiety and depression (HAD) scale and the state–trait anxiety inventory (STAI) to those at weeks 4, 8, 12, 16, and 24. Qualitative participant experience with RebiSmart^®^ was evaluated by assessing the change in score of individual questions as well as defined groups of questions in the patient experience questionnaire (PEQ) at weeks 4, 8, 12, 16, and 24.

### Participants

Adult (18–65 years old) participants with a confirmed diagnosis of RMS according to the 2005 McDonald criteria [23] were recruited from 19 community and academic centres across Canada to participate in MEASURE. Ethics approval was obtained from the institutional review boards at the University of British Columbia (Vancouver, BC) and at each of the other participating centers. Participants were eligible to enrol if they had been prescribed sc IFN β-1a 44 μg tiw according to the Canadian Product Monograph, were registered with the Rebif^®^ Multiple Support Program, and had given written informed consent prior to the study screening period. Participants were excluded from the study if they had (1) been treated with any DMD prior to study enrolment, (2) received any other injectable medication on a regular basis in the week prior to screening and/or during the study, (3) experienced a relapse within 30 days of screening, or (4) a visual or physical impairment that interfered with their ability to self-inject using RebiSmart^®^.

### Data collection and analyses

**Fig. 1:**
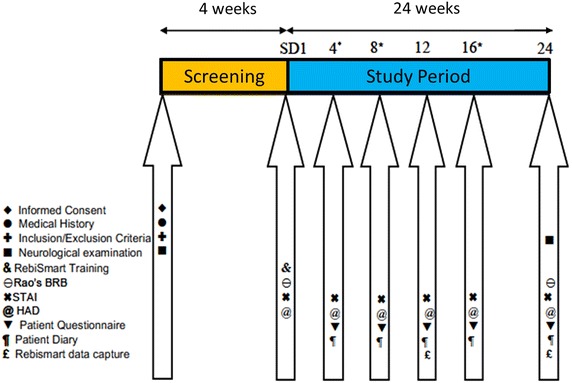
Schedule of study assessments. *Asterisk* assessments at weeks 4, 8, and 16 were done by telephone contact. The current report focuses on data up to week 24 of treatment. *BRB* brief repeatable battery, *HAD* hospital anxiety and depression scale, *SD1* study day 1, *STAI* state–trait anxiety inventory

The schedule of assessments until week 24 is summarized in Fig. 1. During the screening period, demographic data and medical history (including history of MS) were collected. A neurological examination and review of the inclusion/exclusion criteria were also completed. A neurological examination including expanded disability status scale (EDSS) score was completed again at week 24.

Baseline cognitive function was assessed on SD1 using the following four tests from the short form of Rao’s brief repeatable battery (BRB): symbol digit modalities test (SDMT), the selective reminding test (SRT)-Long-Term Storage, the SRT-Consistent Long-Term Retrieval, and the 3-second paced auditory serial addition test (PASAT). These four tests have been previously identified as the most sensitive for detecting cognitive impairment among MS patients [24]. Participants were matched to a normative sample from the Multiple Sclerosis Research Unit at Sunnybrook Health Sciences Centre, using the following variables: age in years, total number of years of education, and sex. The test results from the normative sample were used to create cut-off values for each of the four tests. A failure for each test was defined as the mean minus 1.5 times the standard deviation. Participants were considered cognitively impaired if they failed two or more of the four tests.

Anxiety was assessed on SD1 and at weeks 12 and 24 using the HAD scale and the STAI. The STAI measures temporary, situational anxiety (i.e., state anxiety) and daily, non-situational anxiety (i.e., trait anxiety) [25]. The STAI was administered immediately before injection to get an accurate assessment of the state anxiety associated with injection. The HAD scale is a 14-item scale that determines the level of anxiety and depression experienced by a patient. The total score for the HAD scale anxiety subscale (HADS-A) can range from 0 to 21. We categorized the scores as 0–7 for normal or no anxiety, 8–10 for mild anxiety, 11–14 for moderate anxiety, and 15–21 for severe anxiety. The state and trait subscales of the STAI produce a range of scores between 20 and 80; a higher score indicates greater anxiety. As part of the PEQ, participants were asked to rate their fear of self-injection on SD1 and at weeks 4, 8, 12, 16, and 24. Fear of injection was rated on a scale of 1–5, as follows: not fearful (1), a little (2), moderately (3), a lot (4), and a great deal (5).

The PEQ is a 16-item instrument evaluating both the participant’s experience with RebiSmart^®^ and side effects associated with the administration of Rebif^®^. The PEQ consists of eight questions selected from the Multiple Sclerosis Treatment Concern Questionnaire (MSTCQ) (four pertaining to injection-site reactions and four regarding flu-like symptoms) [22] plus eight questions pertaining to the use of RebiSmart^®^. The full PEQ was administered at weeks 4, 8, 12, 16, and 24. Paired change was evaluated for each individual who responded to a PEQ item at week 4 and a later time point (week 12 or 24).

### Analysis populations

The intent-to-treat (ITT) population comprised 162 participants who were enrolled in the study and had provided written informed consent. The modified ITT population (mITT; n = 158), which comprised all ITT participants who received at least one IFN β-1a injection recorded by the RebiSmart^®^ device, was used for all analyses.

### Statistical analysis

No hypothesis testing was conducted. A total of at least 139 participants were required to provide a 95 % confidence interval (CI) equal to the sample proportion with 5 % precision, based on the hypothesis that 90 % of participants using the auto-injection device for 24 weeks would administer 80 % of the scheduled injections. The 95 % CIs were based on the Agresti–Coull for binomial proportions.

The analytical approach for all endpoints was descriptive in nature. No corrections were made for multiple comparisons because the comparisons were intended to be hypothesis-generating. Nominal p values are presented. Changes from baseline in anxiety measures were analyzed using a two-sided, paired *t* test or a Wilcoxon matched-pairs signed-rank test. Fisher’s exact test (two-sided) was used to compare ≥80 % treatment adherence (yes/no) rates, while the Mann–Whitney U test (two-sided) was used to compare mean treatment adherence between impaired versus non-impaired participants as well as between participants with fear versus no fear of injection. Statistical analyses were performed using the SAS System for Windows, Version 9.2 (Cary, NC).

In addition, a post hoc logistic regression analysis was undertaken to identify potential associations in the mITT population between patient baseline characteristics (gender, cognitive impairment, PASAT, age, education level, months since MS diagnosis, months since MS symptoms, months since last relapse, EDSS, HADS-D, HADS-A, and STAI state and trait anxiety scores) and ≥80 % treatment adherence at week 24. Continuous variables were dichotomized as less than versus greater than or equal to the population median value.

## Results

The baseline demographic and disease characteristics of the 162 participants included in the ITT population are listed in Table 1. Participants had a mean ± SD age of 37.4 ± 9.8 years and were predominantly female (75.3 %) and Caucasian (94.4 %). Nearly all participants (96.3 %) had RMS; the majority of participants (82.1 %) had experienced between 1 and 3 relapses since the onset of MS symptoms, and the mean EDSS score of the study sample was 1.8 ± 1.3. Baseline results of the four cognitive tests constituting a short form of Rao’s BRB showed that 47.8 % of participants failed two or more of the four tests and, by our definition, were classified as cognitively impaired.

**Table 1:** Baseline demographic and disease characteristics of ITT patient population

Characteristic	Result (n = 162)
Demographics	
Age, years	37.4 ± 9.8
Sex	
Male, n (%)	40 (24.7 %)
Female, n (%)	122 (75.3 %)
Race	
Caucasian, n (%)	153 (94.4 %)
Black, n (%)	2 (1.2 %)
Asian, n (%)	2 (1.2 %)
Other, n (%)	5 (3.1 %)
Education, total years completed	14.9 ± 3.3
Disease characteristics	
MS classification	
Relapsing-remitting, n (%)	156 (96.3 %)
Secondary progressive, n (%)	6 (3.7 %)
Months since onset of MS symptoms	53.3 ± 76.1
Months since MS diagnosis	24.0 ± 57.6
Months since last relapse	6.4 ± 9.5
Number of relapses since onset of MS symptoms, n (%)	
0	6 (3.7 %)
1	44 (27.2 %)
2	55 (34.0 %)
3	34 (21.0 %)
4	12 (7.4 %)
≥5	11 (6.8 %)
Expanded disability status scale (EDSS) Score	1.8 ± 1.3
Cognitive function	
Symbol digit modalities test (SDMT)	51.0 ± 13.1
Patients failing test, n (%)	35 (22.9 %)
Selective reminding test (SRT)	
Long-term storage	41.8 ± 15.5
Patients failing test, n (%)	61 (40.4 %)
Consistent long-term retrieval	30.0 ± 15.8
Patients failing test, n (%)	59 (39.1 %)
3-second paced auditory serial addition test (PASAT)	42.8 ± 11.4
Patients failing test, n (%)	47 (31.3 %)
Cognitively impaired (failing ≥2 of the above tests)	67 (47.8 %)

Data presented as mean ± standard deviation except as indicated

*ITT* intent-to-treat, *MS* multiple sclerosis

**Fig. 2:**
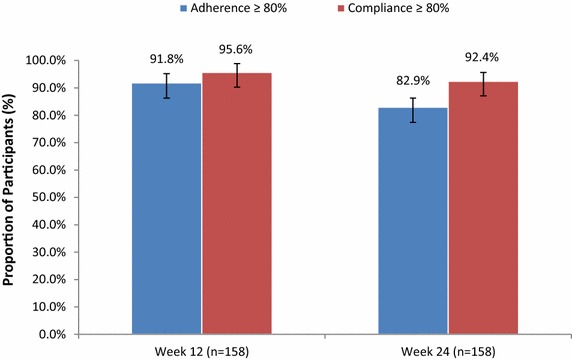
Proportion of participants with ≥80 % treatment adherence and compliance at week 12 and 24 (mITT population). Adherence calculated as 100 × the number of administered injections recorded in the electronic device log divided by the expected number of injections (e.g., 72 injections over 24 weeks). Compliance calculated as 100 times the number of injections recorded in the electronic device log divided by (the number of days on-study) × 3/7. *mITT* modified intent-to-treat

Treatment adherence and compliance rates at weeks 12 and 24 for all participants with at least one injection using the RebiSmart^®^ injector (mITT; n = 158) are shown in Fig. 2. The proportion of participants with ≥80 % adherence to treatment was 91.8 % (95 % CI 86.3, 95.2 %) at week 12 and 82.9 % (95 % CI 76.2, 88.0 %) at week 24. The proportion of participants with ≥80 % compliance to treatment was 95.6 % (95 % CI 91.0, 98.0 %) at week 12 and 92.4 % (95 % CI 87.1, 95.7 %) at week 24. The mean ± SD adherence rate of all participants was 95.7 ± 11.6 % at week 12 and 89.1 ± 20.3 % at week 24. The mean ± SD compliance rate of participants was 97.6 ± 6.9 % at week 12 and 95.2 ± 9.7 at week 24. Only 13 (8.2 %) participants discontinued treatment by the 12th week; by week 24 this number rose to 22 (13.9 %) participants. The reasons for treatment discontinuation at weeks 12 and 24 included participant withdrawal of consent (4 [30.8 %] and 7 [31.8 %], respectively), investigator decision (2 [15.4 %] and 4 [18.2 %], respectively) and other (6 [46.2 %] and 9 [40.95], respectively).

**Fig. 3:**
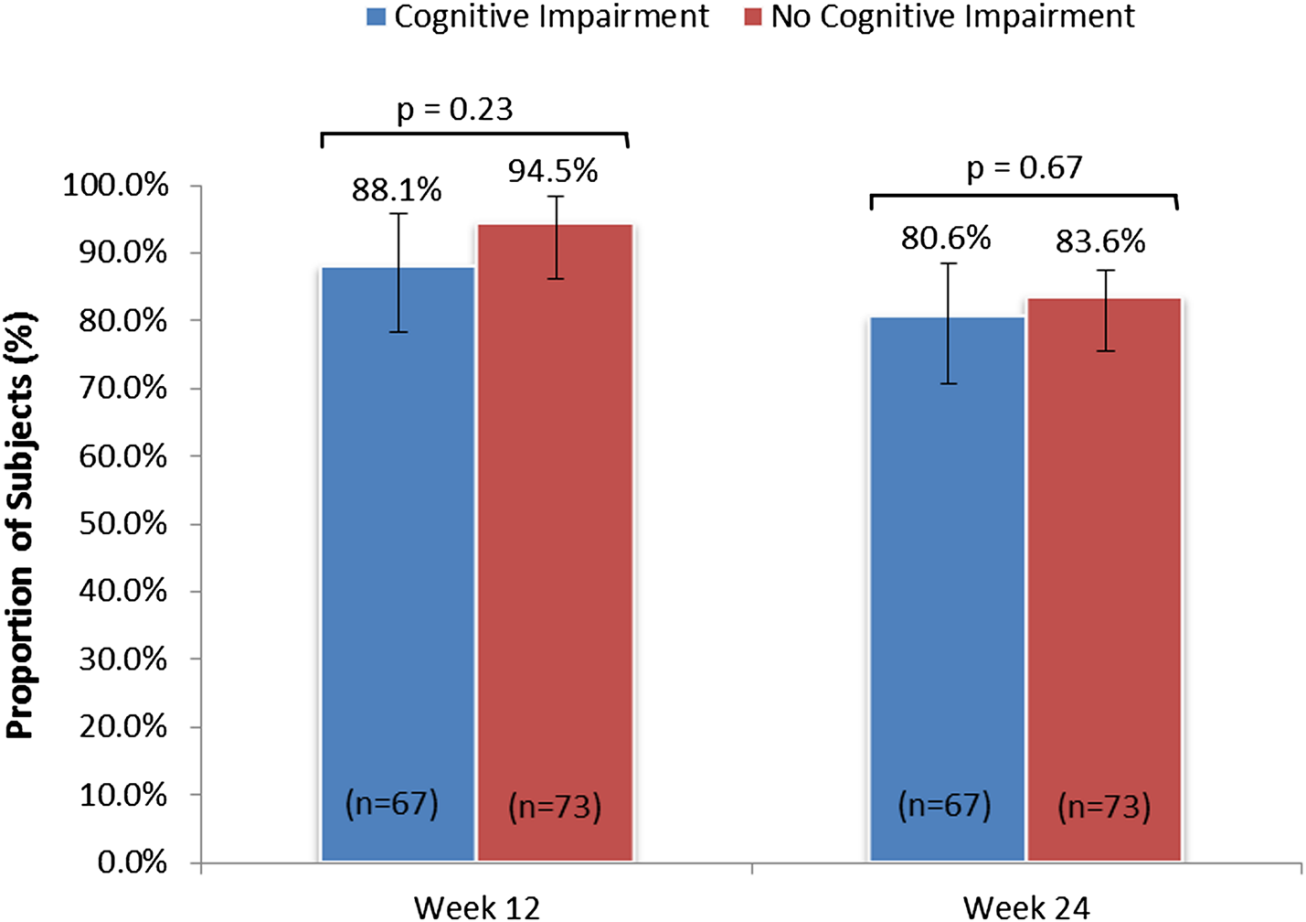
Proportion of participants with ≥80 % treatment adherence according to cognitive impairment at weeks 12 and 24 (mITT population). p value assessed via Fisher’s exact test. *mITT* modified intent-to-treat

Our data (Fig. 3) show that a similar proportion of cognitively impaired and unimpaired participants achieved ≥80 % adherence to treatment at week 12 (88.1 % and 94.5 %, respectively; p = 0.23) and week 24 (80.6 and 83.6 %, respectively; p = 0.67). Similarly, there was no difference in mean adherence rates at week 12 (94.3 ± 14.3 and 96.8 ± 8.7 %, respectively; p = 0.651) and week 24 (86.6 ± 22.9 and 90.4 ± 18.6 %, respectively; p = 0.23) for cognitively impaired and unimpaired participants.

**Fig. 4:**
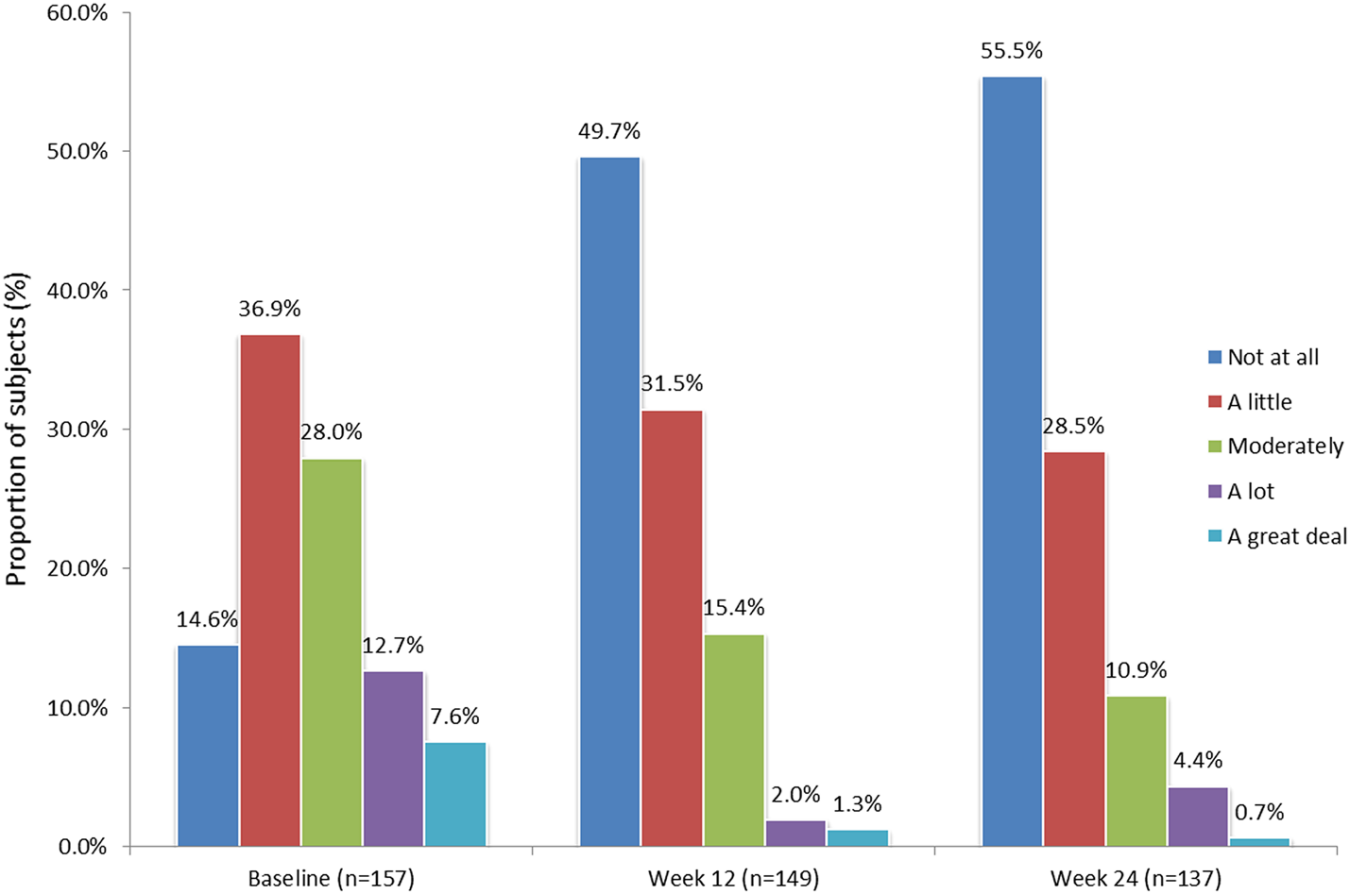
Participant self-reported fear of injection at baseline, week 12, and week 24 (mITT population). Based on patient response to the following question: “Please rate your current fear of self-injection.” *mITT* modified intent-to-treat

**Fig. 5:**
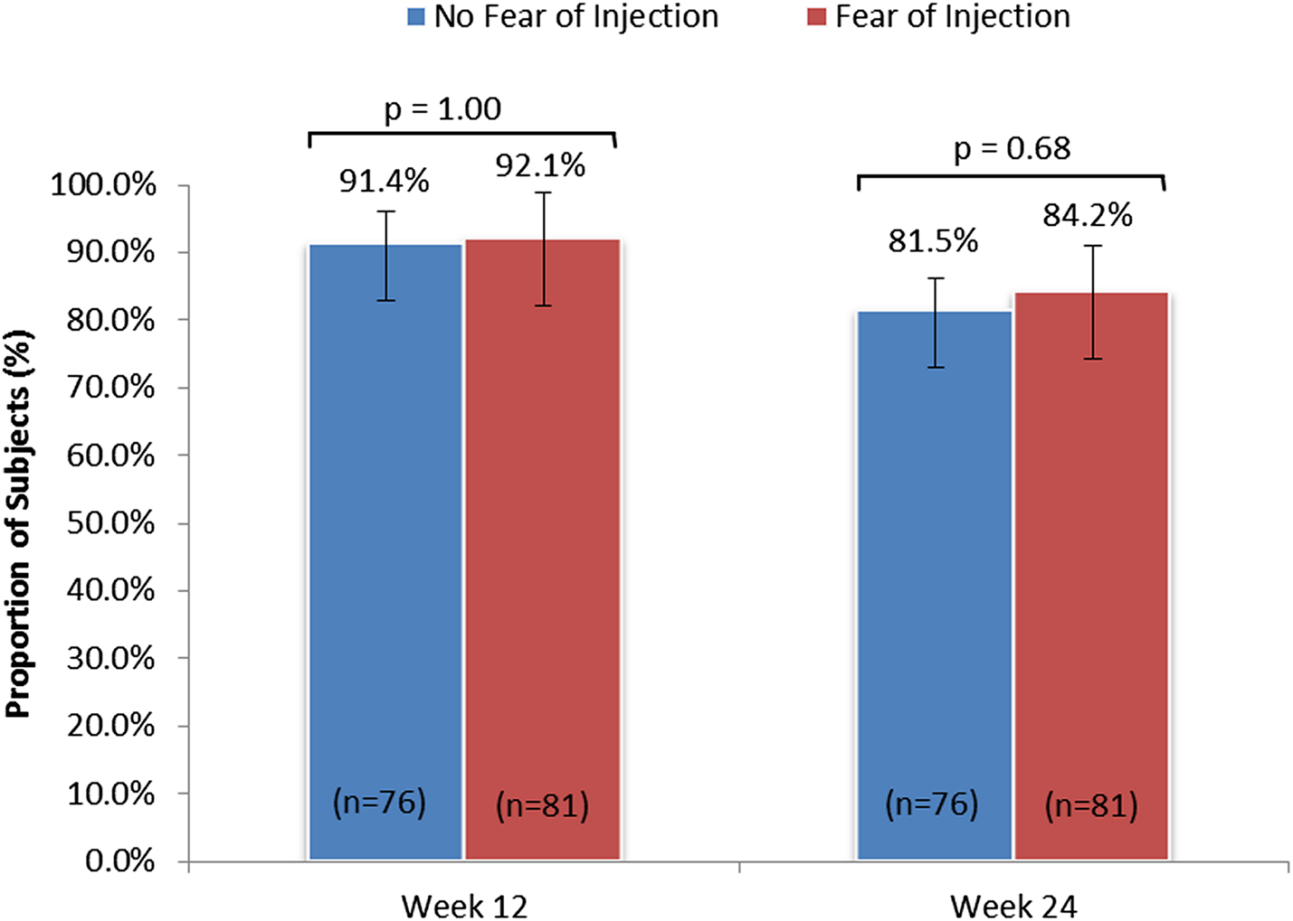
Proportion of participants with ≥80 % treatment adherence according to fear of injection at weeks 12 and 24 (mITT population). p value assessed via Fisher’s exact test. *mITT* modified intent-to-treat

The change in ratings of fear of injection at baseline, week 12, and week 24 are illustrated in Fig. 4. The proportion of participants reporting “moderate,” “a lot,” or “a great deal” of injection fear at baseline was 48.3 %; this number declined to 18.7 % at week 12 and 16.0 % at week 24. Indeed, by week 24, 69.9 % of participants became less fearful of injection than they had been at SD1. Mean ± SD pre-injection state anxiety also declined significantly, from SD1 (40.4 ± 12.3) to week 12 (35.9 ± 12.7; p < 0.0001) and week 24 (36.6 ± 12.9; p < 0.0001). As illustrated in Fig. 5, similar proportions of participants with and without fear of injection at baseline achieved ≥80 % adherence to treatment at week 12 (91.4 % vs. 92.1 %, respectively; p = 1.00) and week 24 (81.5 vs. 84.2 %, respectively; p = 0.68). Mean adherence rates were also similar between participants with versus those without baseline fear of injection at week 12 (96.1 vs. 95.2 %, respectively; p = 0.322) and week 24 (89.0 vs. 89.0 %, respectively; p = 0.901). Indeed, logistic regression querying various patient baseline characteristics identified no significant associations between adherence and patient gender, age, education, cognitive function, disease history, severity of impairment, anxiety or depression (data not shown).

As listed in Table 2, mean ± SD general anxiety, as assessed by the anxiety subscale score of the HAD scale, was significantly lower at weeks 12 and 24 by comparison to baseline (5.8 ± 4.3 and 5.9 ± 4.1 vs. 7.0 ± 4.1, respectively; p < 0.0001 for both). Likewise, mean ± SD trait anxiety was also significantly reduced from baseline to week 12 (38.8 ± 10.9 and 36.7 ± 12.1, respectively; p = 0.002).

**Table 2:** Change in anxiety during treatment (mITT population)

Measure	Baseline	Week 12	Week 24
Hospital anxiety and depression scale			
Anxiety subscale score (range 0–21)	7.0 ± 4.1	5.8 ± 4.3*	5.9 ± 4.1*
State–trait anxiety inventory			
State anxiety subscale (range 20–80)	40.4 ± 12.3	35.9 ± 12.7*	36.6 ± 12.9*
Trait anxiety subscale (range 20–80)	38.8 ± 10.9	36.7 ± 12.1*	37.6 ± 12.1

* p < 0.005 by comparison to baseline, as calculated by a paired t-test

*mITT* modified intent-to-treat

Injection-site reactions (questions 2–5) and flu-like symptoms (questions 10–13) were measured using questions from the MSTCQ at weeks 4, 12, and 24 and are reported in Table 3. The flu-like symptom score declined from a mean score of 11.1 at week 4 to 10.1 at week 24, and the injection-site reaction score increased from 11.2 at week 4 to 12.4 at week 24.

**Table 3:** Multiple Sclerosis Treatment Concern Questionnaire score for subscales relating to injection-site reactions and flu-like symptoms (mITT population)

	Injection-site reaction score		Flu-like symptom score	
	Observed	Paired change	Observed	Paired change
Week 4				
N (missing)	152 (5)	–	152 (2)	–
Mean (SD)	11.2 (3.6)	–	11.1 (4.6)	–
Median (range)	12 (1–20)	–	12 (1–20)	–
Week 12				
N (missing)	149 (2)	144 (7)	148 (3)	143 (8)
Mean (SD)	12.3 (3.0)	1.1 (2.8)	10.8 (4.2)	–0.2 (4.3)
Median (range)	13 (4–20)	1 (−6 to +12)	11 (1–20)	0 (–12 to +17)
Week 24				
N (missing)	137 (1)	133 (5)	137 (1)	133 (5)
Mean (SD)	12.4 (3.0)	1.3 (3.1)	10.1 (4.3)	–0.8 (4.1)
Median (range)	13 (1–18)	1 (−8 to +14)	10 (1–20)	–1 (–3 to +1)

**Fig. 6:**
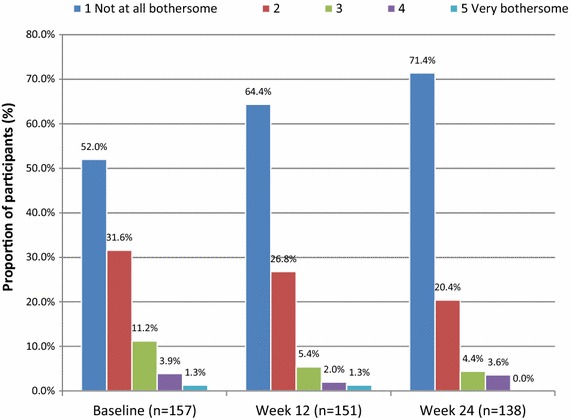
Patient self-reported degree of “bother” associated with RebiSmart^®^ use (mITT population) Based on patient response to the following question: “How bothersome was the amount of time and preparation it took to get everything ready for your Rebif® injection with RebiSmart®?”

Patients’ experience and use of other features of RebiSmart^®^ was evaluated by additional questions within the PEQ. The proportion of participants reporting no pain or mild pain (PEQ question 6) was 53.2 % at week 4, 52.7 % at week 12, and 49.6 % at week 24/early termination. By week 24 or early termination, 63.3 % of participants reported (PEQ question 8) changing the comfort settings on RebiSmart^®^. Of the 42.1 % of participants who had changed the comfort settings by week 4, 50 % reported “a little” improvement in injection experience, 20.3 % reported “moderate” improvement, and 10.9 % reported “a lot” or “a great deal” of improvement. The majority of participants (63.2 %) reported using the device’s injection calendar (PEQ question 9) at week 4 (“a little” [28.3 %], “moderately” [17.1 %], “a lot” [11.2 %], and “a great deal” [6.6 %]); by week 24, 46.7 % of participants continued using this feature. At week 24, nearly all participants (99.3 %) reported that they would like to continue using RebiSmart^®^ as their injector (PEQ question 16). The absence of a visible needle was reported by participants as the most important feature of the RebiSmart^®^ injection device (66.2 %; PEQ question 14), followed by the injection comfort settings for improving injection experience (27.1 %), and lastly the injection calendar for tracking injection administration (6.8 %). Figure 6 shows that 52 % of participants reported the amount of time and preparation of their injection with RebiSmart^®^ was “not at all bothersome” at SD1. That proportion increased to 64.4 % by week 12 and 71.5 % by week 24 or early termination.

## Discussion

RebiSmart^®^ was specifically designed with features to assist patients in maintaining good adherence to treatment so they can realize the full benefits of therapy. The primary objective of the MEASURE study was to evaluate treatment adherence of RMS participants using RebiSmart^®^ for self-injection of sc IFN β-1a at 24 weeks. Our results show that the majority of participants achieved ≥80 % adherence with RebiSmart^®^ and exhibited compliance and discontinuation rates that are superior to other methods of injection for DMDs and very similar to oral DMDs [9, 10, 26]. Additionally, our findings revealed a high level of participant acceptance of the RebiSmart^®^ device.

As objectively recorded by the RebiSmart^®^ dosing log at weeks 12 and 24, 91.8 % (95 % CI 86.3, 95.2 %) and 82.9 % (95 % CI 76.2, 88.0 %) of RMS participants, respectively, achieved good adherence, as defined by administration of ≥80 % of the planned doses. Our adherence data are in agreement with the 12-week, open-label BRIDGE study, which included 119 Italian RMS patients using RebiSmart^®^ for self-injection of sc IFN β-1a [27]. In that trial, 88.2 % of patients achieved good treatment adherence over a 12-week period; medical reasons and forgetfulness were the main causes of missed doses. This level of adherence compares favourably with the estimated 61–75 % of MS patients who self-report being adherent to traditional injectable DMD therapy [9, 10], as well as the 58.5–62.3 % of MS patients who regularly refill their prescriptions for injectable DMD therapy over the long-term [8, 11]. Additionally, the level of adherence with RebiSmart^®^ reported herein is close to that reported for oral medication use among patients with other chronic diseases (93.0 %) [26].

The proportion of participants with ≥80 % compliance to treatment was 95.6 % at week 12 and 92.4 % at week 24; these rates also compare favourably with compliance ≥80 % at week 12 among 96.3 % of patients in the BRIDGE study. Furthermore, only 13.9 % of participants had discontinued treatment with RebiSmart^®^ by week 24, by comparison to an estimated 20.9–26.4 % of MS patients who discontinue DMD therapy within 6 months of treatment initiation [12]. Based on comparisons with available literature, MS treatment with RebiSmart^®^ appears to produce superior adherence and persistence rates relative to other injection methods.

Several features of RebiSmart^®^ may contribute to the high level of treatment adherence and compliance documented in this study. Approximately half of participants (48.4 %) reported some degree of fear of injection at baseline, a known risk factor for poor adherence [9, 28]. In order to overcome this common treatment barrier, the RebiSmart^®^ auto-injector employs a concealed needle that cannot be seen by patients before or after the injection. Additionally, in order to reduce potential injection-site pain, the device includes an adjustable injection comfort setting that allows for adjustment of needle speed, injection speed, depth, and duration. Participants in the current trial identified the absence of a visible needle as the most important benefit of the RebiSmart^®^ auto-injector, followed by the comfort settings for improving injection experience. By weeks 12 and 24, two-thirds (66.9 and 69.9 %, respectively) of RMS participants became less fearful of injection than they had been at baseline. Additionally, participants’ baseline anxiety level prior to injection was also reduced by weeks 12 and 24 of treatment (from 40.4 ± 12.3 to 35.9 ± 12.7 and 36.6 ± 12.9, respectively). Interestingly, treatment adherence at weeks 12 and 24 was equally high among participants who had expressed anxiety at baseline compared with those who had not (96.1 vs. 95.2 % at week 12 and 89.0 vs. 89.0 % at week 24). Our data suggest the hidden needle and injection comfort settings may contribute to reducing injection fear and anxiety and maintaining good adherence and compliance.

MS affects several aspects of cognitive function including processing speed, executive function, and memory [16, 29]. Here, 47.6 % of RMS patients had some degree of cognitive dysfunction at baseline. The proportion of participants with cognitive dysfunction in our sample is in general agreement with previously reported estimates in this population (40–65 %) [16]. Forgetfulness, a dimension of cognitive impairment, has been previously identified as a major reason for poor adherence to MS therapy [9]. The injection calendar and an optional injection reminder alarm are RebiSmart^®^ features designed to overcome forgetfulness. We did not observe an influence of cognitive impairment on treatment adherence. Cognitively impaired and non-impaired RMS participants achieved similar rates of mean adherence to sc IFN β-1a administered with RebiSmart^®^ at weeks 12 (94.3 ± 14.3 and 96.8 ± 8.7 %, respectively) and 24 (86.6 ± 22.9 and 90.4 ± 18.6 %, respectively). This finding agrees with the BRIDGE study, which reported no association between cognitive function, as assessed by PASAT score, and adherence to RebiSmart^®^ sc IFN β-1a treatment at 12 weeks [27]. These findings suggest features of RebiSmart^®^ may help participants overcome the barrier of cognitive impairment and achieve good adherence and compliance.

Objective adherence monitoring using RebiSmart^®^ may also contribute to improved treatment adherence for participants. While self-report is a convenient method for ascertaining treatment adherence, MS participants tend to overestimate their level of adherence [30]. Thus, treatment failure due to poor adherence may be misinterpreted by physicians as a lack of treatment efficacy, leading to unnecessary dose escalation or treatment change. RebiSmart^®^ is the first injection device used in MS that provides an accurate record of dosing history. While the dosing history log was rated by participants in an earlier study as the least useful feature of the RebiSmart^®^ device [31], this function can be of great utility to physicians. The log, which can be readily downloaded onto the physician’s computer, provides an up-to-date, objective measure of a participant’s treatment adherence, thereby overcoming the limitations inherent in patient self-report.

Our findings also revealed a high level of acceptance of the device, with almost all participants (99.3 %) reporting at 24 weeks that they would like to continue using RebiSmart^®^ as their injector. This observation agrees with prior patient rating studies that showed RebiSmart^®^ is generally well-accepted by RMS patients [22, 31]. For instance, a prior multicentre, open-label, observational, phase IV study in the UK and Ireland observed that 92 % of RMS patients liked using the device. Convenience and ease of use have been frequently reported by RMS participants as the major benefits of RebiSmart^®^ [22, 31]. In the current study, 71.5 % of participants indicated that injection with RebiSmart^®^ was convenient.

The current study has a number of limitations. As the MEASURE study lacked a control or active comparator group, it is not possible to generalize the current findings beyond the population of patients receiving sc IFN β-1a via the RebiSmart^®^ injection system. In addition, since p values in our exploratory analyses were not corrected for multiple comparisons, the statistical significance of our secondary outcomes should be interpreted with caution. Finally, while 24 weeks of follow-up currently represents the longest report of treatment adherence, longer-term follow-up data from the MEASURE study, extending to 96 weeks of treatment, is forthcoming.

## Conclusions

The RebiSmart^®^ auto-injection device addresses a number of known barriers to MS treatment adherence and is well received by RMS patients. The use of an electronic auto-injector for administration of IFN β-1a is associated with good treatment adherence at 6 months among four in five RMS patients, regardless of cognitive function or fear of injection at baseline. Future analyses from this trial will determine whether these encouraging adherence rates correspond with a reduction in the frequency of relapse and a delay in disability progression.
